# Genome sequencing-based discovery of a novel deep intronic APC pathogenic variant causing exonization

**DOI:** 10.1038/s41431-023-01322-y

**Published:** 2023-02-24

**Authors:** Anikó Bozsik, Henriett Butz, Vince Kornél Grolmusz, Csaba Polgár, Attila Patócs, János Papp

**Affiliations:** 1 Department of Molecular Genetics, National Institute of Oncology, Ráth György út 7-9, Budapest, H-1122 Hungary; 2 Hereditary Cancers Research Group, Hungarian Academy of Sciences - Semmelweis University, Nagyvárad tér 4, Budapest, H-1089 Hungary; 3 National Tumorbiology Laboratory, National Institute of Oncology, Ráth György út 7-9, Budapest, H-1122 Hungary; 4 Center of Radiotherapy, National Institute of Oncology, Ráth György út 7-9, Budapest, H-1122 Hungary; 5 Department of Oncology, Semmelweis University, Ráth György út 7-9, Budapest, H-1122 Hungary

**Keywords:** Cancer genetics, Cancer genetics

## Abstract

Familial adenomatous polyposis (FAP) is a hereditary cancer syndrome that occurs as a result of germline mutations in the *APC* gene. Despite a clear clinical diagnosis of FAP, a certain proportion of the *APC* variants are not readily detectable through conventional genotyping routines. We accomplished genome sequencing in duo of the disease-affected proband and non-affected sibling followed by in silico predictions and a series of RNA-based assays clarifying variant functionality. By prioritizing variants obtained by genome sequencing, we discovered the novel deep intronic alteration APC:c.531 + 1482 A > G that was demonstrated to cause out-of-frame exonization of 56 base pairs from intron 5 of the gene. Further cDNA assays confirmed, that the aberrant splicing event was complete and its splice product was subject to nonsense-mediated decay. Co-segregation was observed between the variant carrier status and the disease phenotype. Cumulative evidence confirmed that APC:c.531 + 1482 A > G is a pathogenic variant causative of the disease.

## Introduction

Familial adenomatous polyposis (FAP) is an autosomal dominantly inherited polyposis subtype of colorectal cancer diseases, accounting for 0.5–1% of all colorectal cancer cases [1]. FAP has a strong genotype correlation with the susceptibility locus adenomatous polyposis coli (*APC*), with a germline mutation detection rate of 60–90% [2, 3]. The missing heritability can be largely explained by the presence of certain mutation types of the *APC* gene that are outside the scope of routine genotyping methods. Such mutations include promoter variants [4], copy-neutral inversions [2], or deep intronic variants [5]. However, detection of the causative genetic elements may be critical to the selection of appropriate therapies, preventive measures and genetic counseling of the affected family members. Here, we disclose a mutation detection history that unravels a novel deep-intronic *APC* pathogenic variant in a polyposis family meeting clinical criteria of FAP. Our investigation demonstrates the utility of whole genome sequencing (WGS) for the discovery of mutations beyond exonic regions.

## Methods

### Family selection and germline genetic screening

A classical FAP family with adenomatous polyposis affecting multiple members over three generations was assigned for genetic analysis at the Department of Molecular Genetics, National Institute of Oncology, Hungary. DNA was extracted from blood leukocytes using the Gentra DNA Blood extraction Kit (Qiagen, Hilden, Germany). Bidirectional Sanger sequencing of the coding exons and exon-intron boundaries of the *APC* gene and multiplex ligation-dependent probe amplification (MLPA) testing for copy number variants by P043-E1 kit (MRC-Holland, The Netherlands) were performed. Whole exome sequencing was done as previously described [6, 7] (see Supplementary Methods). Whole genome sequencing (WGS) was designed as a *duo* of the disease-affected proband and his non-affected brother (details in Supplementary Methods). The resulting variant list was prioritized for colon polyposis genes [8] and only considered changes with a frequency of less than <0.01 in gnomAD database and present only in the affected family member. All participants gave written informed consent for the genetic testing.

### Computational predictions

The filtered WGS variant list was subjected to in silico splice prediction analyses using SpliceAI (https://spliceailookup.broadinstitute.org) and scores >0.2 were evaluated. Splicing regulatory elements were queried by HSF v.3.1 (https://www.genomnis.com/access-hsf). Pathogenicity of the dedicated variant was ascertained by ACMG’s qualitative rules [9]. PP3 (Score: +1), PP4 (Score: +1), PP1 (Score: +1) PM2 (Score: +1), PVS1 (Score: +8). Pathogenicity was assumed, if the total score was ≥10.

### RNA-based testing

Total RNA was isolated from peripheral blood using Tempus Spin RNA Isolation Kit (ThermoFisher Scientific). RNA was reverse transcribed with Protoscript II First Strand Synthesis Kit (New England Biolabs, MA, USA) using random hexamers. PCR reactions from the cDNA template (RT-PCR) were carried out using Multiplex PCR Kit (Qiagen). RT-PCR products were visualized by electrophoresis on 1.5% agarose gels and on DNA1000 chips (Agilent Technologies, CA, USA). Allele imbalance test and completeness of aberrant splicing were done as previously reported [10]. Fluorescent fragment analysis was performed as follows: 24 cycles of duplex PCR reactions were carried out with Multiplex PCR Kit (Qiagen) using forward primers designed to selectively amplify the normal and the aberrant transcripts, respectively, paired with a FAM-labelled reverse primer in one reaction. The PCR products were subjected to capillary electrophoresis on an ABI3500 Genetic Analyzer (ThermoFisher Scientific) and visualized with Gene Mapper Software v.6. (Primers are listed in Supplementary Table).

## Results

### Genotyping

**Fig. 1:**
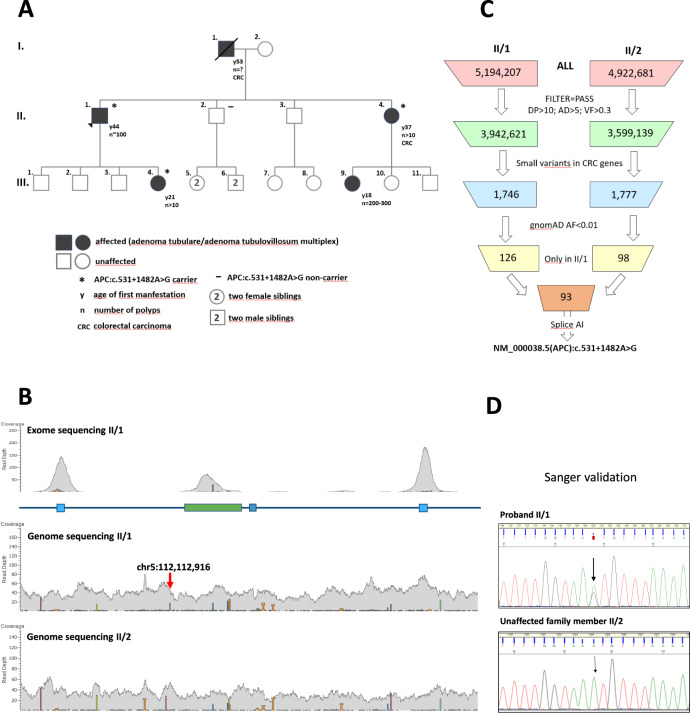
Germline genetic screening. **A** Pedigree of the tested FAP family. Proband II/1 is marked with black triangle. **B** Snapshot of the binary alignments of the sequenced reads visualized with Golden Helix data analysis software (Golden Helix, MT, USA). The presented genetic slice stretches from exon 5 to exon 6 of the *APC* gene on the reference genome build GRCh37/hg19. The upper scene shows exome sequencing results of the II/1 proband. The lower scenes present genome sequencings of the family members II/1 and II/2 respectively. Blue and green boxes are exonic regions. Colored bars are diverse variant positions. Red arrow highlights the exact genomic position of the variant selectively present in II/1. **C** Prioritization scheme of the WGS variants. Upmost initial boxes show the number of all genotyped variants. The variant numbers of the successive levels are calculated according to the indicated selection criteria. DP position depth, AD allele depth, VF variant allele frequency, CRC colorectal cancer, AF allele frequency. **D** Sanger sequencing validation of NM_000038.5(APC):c.531 + 1482 A > G genotyped by WGS. The proband was a heterozygote carrier of the variant, whereas the unaffected family member was non-carrier. Black arrows indicate the variant positions.

A family with a clinical diagnosis suggestive of classic FAP was enrolled for germline genetic testing. Several family members spanning 3 generations were affected with polyposis-type colorectal cancer, with 10–300 adenomatous polyps at diagnosis, thus meeting the requirements for the syndrome and being eligible for genetic counseling according to the relevant guidelines [11]. Age of onset of the disease ranged from 18 to 53 years (pedigree is shown in Fig. 1A). Genomic DNA from the proband (II/1) was tested by Sanger sequencing for all coding exons and exon-intron boundaries of the *APC* gene. MLPA testing for large copy number variations of the gene was also performed. No pathogenic variant or large deletions/duplications were identified. Subsequently, whole exome sequencing (WES) was carried out to screen for potentially deleterious variants in established polyposis genes [8]. Again, no clinically relevant variant was recognized. Subject II/1 then underwent WGS, the most comprehensive type of genotyping, along with his unaffected sibling (II/2) who did not develop disease until age 55. Resulting variants with adequate quality metrics called within the polyposis genes were listed, filtered for only rare variants (<0.01 in gnomAD database) and evaluated only those, present in the proband but absent from the healthy relative (Fig. 1B). Annotations with in silico splice prediction softwares revealed a deep intronic variant with potential splicing effect (hg19)chr5:112112916 A > G; NM_000038.5(APC):c.531 + 1482 A > G (prioritization workflow is shown in Fig. 1C). Confirmatory Sanger sequencings were performed (Fig. 1D). This variant was novel, not registered in the Variant Database of Hereditary Gastrointestinal Tumors (http://insight-database.org/genes/APC) and absent in large population screenings (gnomAD https://gnomad.broadinstitute.org, 1000 Genomes https://www.internationalgenome.org or ExAC http://exac.broadinstitute.org). SpliceAI algorithm predicted (score: 0.93) this base change to activate a splice donor site five nucleotides upstream. Human Splice Finder revealed, that the variant affected several intronic splice silencer motifs (Supplementary Figure). The variant tested positive in two additional family members (II/4 and III/4), both affected with the disease, whereas it was not present in the healthy family member II/2 (current age: 55 years).

### RNA-based testing

**Fig. 2:**
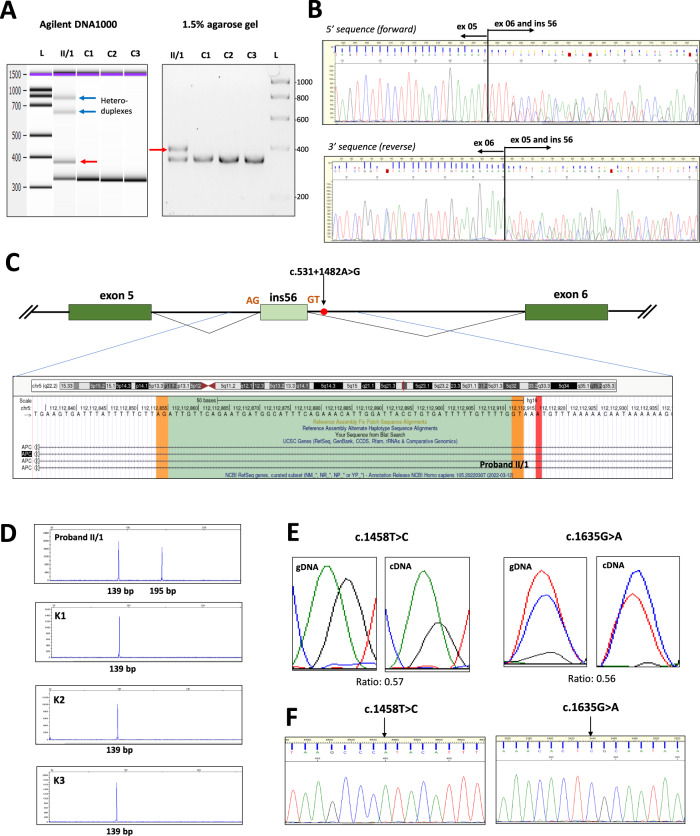
Effect of the variant c.531 + 1482 A > G on transcript level. **A** RT-PCR product of the variant carrier yielded a larger extra band on 1.5% agarose gel electrophoresis. The same result is seen on Agilent DNA1000 chip. Black arrow points out the band for the normal transcript, red arrow indicates the larger band of the aberrantly spliced transcript and blue arrows show the heteroduplexes. L: ladder, C1, C2, C3: controls. **B** Sanger sequencing of the RT-PCR amplicon revealed a 56-bp exonization of intron 5 of *APC*. Forward sequence and reverse sequence superpositions show the 5’ and the 3’ ends of the exonized stretch, respectively. **C** Graphical representation of the exonization of a 56-bp stretch of intron 5 of *APC* as a result of the germline variant c.531 + 1482 A > G. Red dot shows the germline intronic base change, dark green boxes are canonical exons and light green box is the exonized intronic region. Orange AG and GT dinucleotides are the cryptic donor and acceptor sites activated as a result of the variant. The exonized region sequence is highlighted below, visualized by a screenshot of the UCSC Genome Browser (https://genome-euro.ucsc.edu). Exon and intron sizes are not to scale. **D** Fluorescent capillary electrophoresis performed by selective amplification of the normal and aberrantly spliced products. The 195 bp peak is consistent with the aberrant fragment and is present exclusively in the variant carrier. **E** Allelic imbalance measured at the positions of coding heterozygote variants c.1458 T > C and c.1635G > A of the proband by comparing electrophoretic peak height ratios of variant and reference signs in cDNA and gDNA. Calculated ratios are indicated below the figures (see Methods for calculation formula). The variant positions are depicted from their reverse sequences. **F** Tagging SNP test carried out for the detection of the degree of the aberrant splice event. The RT-PCR products are amplified selectively from the wild-type transcript. The arrows point out the positions of the exonic germline heterozygote variants and indicate, that only the reference nucleotide is present at these positions at the RNA-level, therefore the normal transcript comes from only one allele.

RNA was obtained from both the proband (II/1) and another affected relative of the family (III/4) who was also heterozygote for the variant. RT-PCR from the cDNA was carried out with primers designed for exons surrounding the variant. Gel electrophoresis demonstrated the presence of the same aberrant product for both carriers (Fig. 2A). Bidirectional Sanger sequencing confirmed that it was a 56-bp exonization from intron 5 (Fig. 2B) as a result of the activation of a pre-existing cryptic GT donor site five nucleotides upstream of the variant (Fig. 2C). The activated donor site induced a nearby AG dinucleotide as a functional acceptor site and these together established an extra exon from the intronic sequence (NM_000038.5(APC):r.531_532ins531 + 1422_531 + 1477). The pseudoexon was out-of-frame, and the inferred translated product was p.(Phe178Ilefs14*). The private existence of the aberrantly spliced product in variant carriers was certified by fluorescent fragment analysis (Fig. 2D).

The possible nonsense-mediated decay (NMD) effect on the aberrant splice product was tested by allelic imbalance using exonic heterozygote variant positions as markers. Only the proband (II/1) was eligible for this test because he was heterozygous for c.1458 T > C (rs2229992) and c.1635G > A (rs351771). Relative allelic area under the curve ratios measured at the heterozygote positions were 0.57 and 0.56, respectively (Fig. 2E), indicating that the transcript carrying the minor alleles was reduced by about half. The III/4 family member was genotyped homozygous for both polymorphic variants, providing indirect evidence that c.531 + 1482 A > G variant is in phase with the minor alleles of these markers. This fact is consistent with the allelic imbalance result: the allele, carrying also c.531 + 1482 A > G was detectable in lower quantity.

Next, we addressed the integrity of the aberrant splicing: long RT-PCR was performed using primers designed to amplify only the normal-length wild-type transcript, and quantified the allelic composition of the product by examining tagging positions. Sanger sequencing pointed out the exclusive presence of the major alleles at both tagging polymorphic positions rs2229992 and rs351771 (Fig. 2F), indicating that the normal transcript was not admixed by the variant-carrier allele. This outcome confirmed the completeness of the aberrant splicing event.

## Discussion

The selected family showed obvious clinical features of classical FAP and the appearance of the disease in several generations indicated the conclusive presence of a heritable factor and ruled out mosaicism [12]. However, routine genetic testing failed to detect a causative germline susceptibility variant in the *APC*. Therefore, we performed two further sequencing sessions with increasing comprehensiveness. WES also did not point out possible pathogenic mutations in various polyposis genes. Finally, WGS provided a solution to the conundrum of the disease inheritance. Computational prioritization of the WGS variants highlighted a novel deep intronic alteration NM_000038.5:c.531 + 1482 A > G. Functional characterizations on the cDNA level discovered that the variant elicited an out-of-frame exonization. The aberrant splicing event was complete, which is critical in terms of pathogenicity [13]. The aberrant transcript was only present in variant carriers and the variant co-segregated with the disease. The variant-containing transcript was partially degraded, presumably by NMD. Based on the summed score of these pieces of evidence (score: 12), this genetic alteration was judged as a genuine pathogenic variant by the ACMG guidelines [9]. Diverse pseudoexon formations due to deep intronic variants have been described in the *APC* gene by others [14, 15], most of them were infrequent findings reported only in single families. Approximately 15–20% of the total germline *APC* pathogenic variants emerge de novo [16], so isolated and diverse pathogenic hits can be attributable to independent germline mutation events of this gene. So far, WGS-based discovery of causative mutations was reported in various clinical syndromes, possessing strong phenotype-genotype correlations [17–20]. Performing *APC* whole gene sequencing or even WGS is especially valid for multi-generational FAP families, if panel sequencing results are negative. Comparison of the variants of affected and non-affected family members promotes clinical evaluation.

## Supplementary information

Supplementary material

## Data Availability

The variant was deposited in the LOVD database with accession number ♯00426064. (https://databases.lovd.nl/shared/individuals/00426064).
