# Supplementary material for: Genome sequencing-based discovery of a novel deep intronic APC pathogenic variant causing exonization
Source: Eur J Hum Genet. 2023 Feb 24;31(7):841–5. doi: 10.1038/s41431-023-01322-y (PMC10326037; doi:10.1038/s41431-023-01322-y)

Supplementary Methods

Whole Exome Sequencing (WES)

Twist Human Core Exome library preparation with a Twist mitochondrial panel (Twist Bioscience, CA, USA) was used for target enrichment, and sequencing was performed on NovaSeq Illumina platform (Illumina, CA, USA) with an average coverage of 100x. Data were analyzed by applying the Genome Analysis Toolkit (GATK, https://gatk.broadinstitute.org) germline short variant discovery (SNPs + Indels) algorithm applying the best users practice. Sequence and short copy number alterations of 23 genes related to hereditary colon polyposis and colon carcinoma was regarded: *APC, AXIN2, BMPR1A, CDH1, EPCAM, FAF1, GREM1, MLH1, MLH3, MSH2, MSH3, MSH6, MUTYH, NTHL1, PMS2, POLD1, POLE, PTEN, RNF43, SMAD4, STK11, TGFBR2, TP53* (gene list is selected according to NCCN colon guidelines complemented with genes involved in colon cancer mutation spectrum based on literature knowledge). Whole coding region and ±5 bp in each exon of the genes was evaluated. The coverage was an average of 208 reads/base (min: 47/base).

Whole Genome Sequencing (WGS)

Whole genome sequencing was designed as *duo* of the disease-affected proband and his non-affected brother using NEBNext Ultra II FS DNA Library Prep Kit for Illumina (New England Biolabs, MA, USA). Sequencing was run on Illumina NovaSeq 6000 instrument (Illumina, CA, USA). Data analysis was performed using Illumina BaseSpace Dragen Germline 3.8.4. application with default settings. hg19 build was applied as human reference sequence (https://genome-euro.ucsc.edu). Sequencing quality metrics: 99.93% of the *APC* gene region was covered with ≥10 reads and 96.45% of the gene was covered with ≥20 reads.

Allele imbalance test and completeness of aberrant splice

Allele imbalance was tested at two exonic positions, c.1458T>C (rs2229992) and c.1635G>A (rs351771), which were genotyped heterozygous in the gDNA of the proband. Genetic segment overlapping the variants was amplified from the cDNA of the proband with primers 5'-GGACTACAGGCCATTGCAGA-3' and 5'-TTGTAGCTATCAAGCTGGACACA-3'. After Sanger sequencing, the electropherogram peak intensities at the heterozygote positions were compared, as area under the curve (AUC) ratios. The ratios of the cDNA were normalized to that of the gDNA. Calculation formula: ($AUC_{reference\ in\ gDNA}$ / $AUC_{variant\ in\ gDNA}$) : ($AUC_{reference\ in\ cDNA}$ / $AUC_{variant\ in\ cDNA}$). Splicing completeness was tested harnessing the same polymorphisms c.1458T>C (rs2229992) and c.1635G>A (rs351771) as tagging variants. Shortly, RT-PCR amplicon comprising the tagging variants was selectively amplified from the wild type transcript using primers 5'-AGATAGTCTTCCTTTAACTGAAAATTTTT-3' and 5'-TTGTAGCTATCAAGCTGGACACA-3'. Sanger sequencing electropherogram peaks for the variant positions were queried. Any sign of peak superposition is a marker of incomplete splicing, i.e. the involvement of the other allele in the normal splice product.

Supplementary Table

Sequences of the primers applied for DNA and RNA tests

Name	Sequence	Application
APC-C2for	ATGTCCCTCCGTTCTTATGG	aberrant splice detection
APC-C2rev	TTGTTCTTCCATCGCAACTC	aberrant splice detection
APC-C3rev	CTTGGTTCCCAGATGACTTG	aberrant splice detection
APC_C-ex11_For	GGACTACAGGCCATTGCAGA	alleleic imbalance
APC_C-ex14_Rev	TTGTAGCTATCAAGCTGGACACA	alleleic imbalance
APC_aberr_F	AGATAGTCTTCCTTTAACTGAAAATATTG	fluorescent fragment analysis, aberrant splice measure
APC_wild_F	AGATAGTCTTCCTTTAACTGAAAATTTTT	fluorescent fragment analysis
APC_cDNS_ex6_R_FAM	CTGTGCTCGTTTTTCCATATC	fluorescent fragment analysis
APC_C-ex14_Rev	TTGTAGCTATCAAGCTGGACACA	aberrant splice measure
APC_int05_F	5'-AACTTGAAATGTATGACTTAAGGAAAT-3'	germline validaton
APC_int05_R	5'-TTCTCATATGCCTAAGGACCA-3'	germline validaton

Supplementary Figure

Human Splicing Finder track analysis for the chromosome segment hg19 chr5:112,777,183_112,777,243. Turquoise bar points out the variant position. Green dot denotes donor site, yellow dot with green margin indicates new cryptic donor site. ESE: predicted splicing enhancer elements ESS: predicted splicing silencer elements ESR: predicted splicing regulation profile.

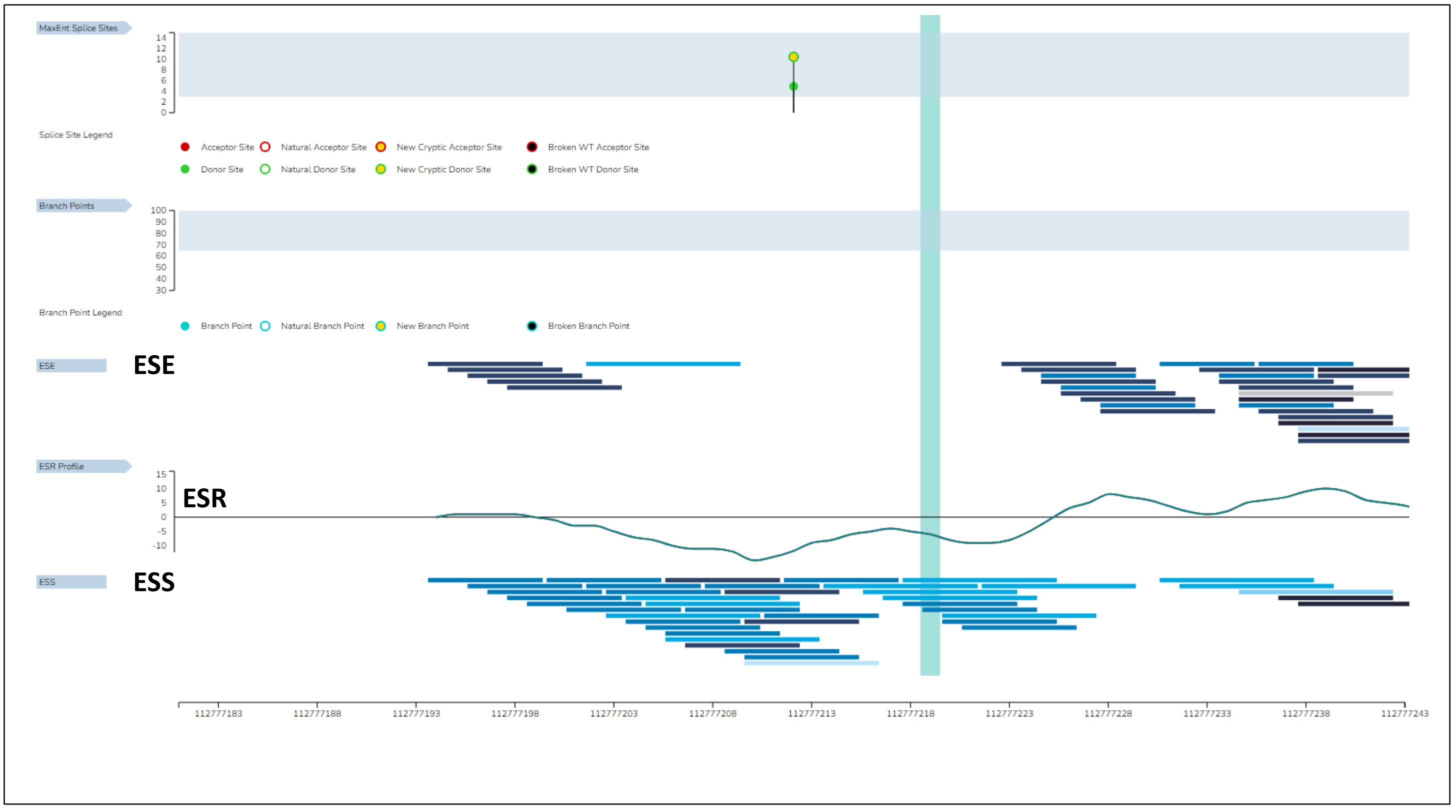

Supplement: Supplementary file 1 — Supplementary material [file 41431_2023_1322_MOESM1_ESM.pdf]
